# Cyanophycin Modifications—Widening the Application Potential

**DOI:** 10.3389/fbioe.2021.763804

**Published:** 2021-10-19

**Authors:** Natalia Kwiatos, Alexander Steinbüchel

**Affiliations:** International Center for Research on Innovative Biobased Materials (ICRI-BioM)—International Research Agenda, Lodz University of Technology, Lodz, Poland

**Keywords:** cyanophycin, multi-L-arginyl-poly-l-aspartate, polyaminoacid, biopolymer, chemical and enzymatic modifications

## Abstract

A circular bioeconomy approach is essential to slowing down the fearsome ongoing climate change. Replacing polymers derived from fossil fuels with biodegradable biobased polymers is one crucial part of this strategy. Cyanophycin is a polymer consisting of amino acids produced by cyanobacteria with many potential applications. It consists mainly of aspartic acid and arginine, however, its composition may be changed at the production stage depending on the conditions of the polymerization reaction, as well as the characteristics of the enzyme cyanophycin synthetase, which is the key enzyme of catalysis. Cyanophycin synthetases from many sources were expressed heterologously in bacteria, yeast and plants aiming at high yields of the polymer or at introducing different amino acids into the structure. Furthermore, cyanophycin can be modified at the post-production level by chemical and enzymatic methods. In addition, cyanophycin can be combined with other compounds to yield hybrid materials. Although cyanophycin is an attractive polymer for industry, its usage as a sole material remains so far limited. Finding new variants of cyanophycin may bring this polymer closer to real-world applications. This short review summarizes all modifications of cyanophycin and its variants that have been reported within the literature until now, additionally addressing their potential applications.

## Introduction

**FIGURE 1:**
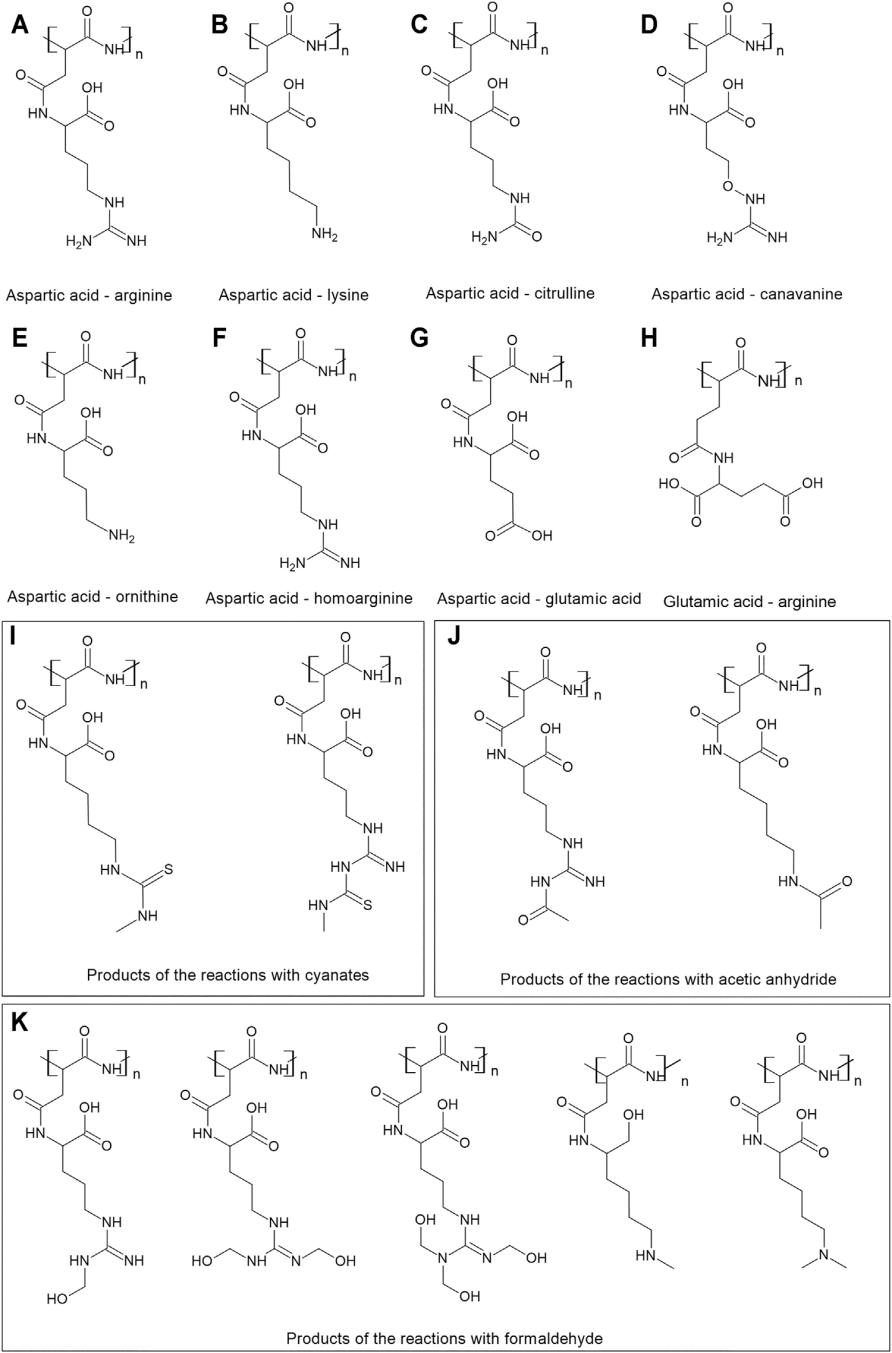
Cyanophycin and its modifications. **(A)** The basic structure of cyanophycin–aspartic acid in the backbone and arginine in the side chain; the next drawings present cyanophycin with aspartic acid in the backbone and different side chains: **(B)** lysine, **(C)** citrulline, **(D)** canavanine, **(E)** ornithine, **(F)** homoarginine, **(G)** glutamic acid. **(H)** glutamic acid in the backbone and arginine in the side chain. The drawings I-K presents the structures of the products of the reactions of cyanophycin with **(H)** cyanates, **(I)** acetic anhydride **(J)** formaldehyde.

Cyanophycin, also referred to as cyanophycin granule polypeptide or multi-L-arginyl-poly-l-aspartate, is a non-ribosomally synthesized amino acid polymer found in cyanobacteria and some heterotrophic bacteria. It serves microorganisms as a temporary storage compound primarily for nitrogen, but also for energy and carbon (Merritt et al., 1994; Li et al., 2001; Frommeyer et al., 2016). It is composed of aspartic acid in the backbone and arginine residues as the side chains (Figure 1 Figure 1A). Typically, these amino acids are present in equimolar amounts. Cyanophycin is synthesized by the enzyme cyanophycin synthetase (CphA), which requires a primer, Mg^2+^, K^+^, ATP and two amino acids for its catalytic action (Simon, 1976). CphA uses a short peptide as a primer and then extends it to produce cyanophycin (Berg et al., 2000; Sharon et al., 2021). The α-carboxylic group of aspartic acid in the backbone is activated by phosphorylation, whereby ATP is converted to ADP. Then, the next aspartic acid is bound by peptide bond to the C-terminus of the backbone. Next, the γ-carboxylic acid is activated by phosphorylation and arginine is attached at this position by an isopeptide bond (Forchhammer and Watzer, 2016; Frommeyer et al., 2016; Du et al., 2019).

Cyanophycin occurs mainly as an insoluble inclusion in the cytoplasm, but is also soluble under certain conditions. Cyanophycin is a polydisperse polymer with molecular masses between 25 and 100 kDa (Frommeyer et al., 2016; Du et al., 2019). The molecular mass range for insoluble cyanophycin is approximately between 20 and 25 kDa, while for soluble cyanophycin this value lies between 8 and 20 kDa. Regular cyanophycin is regarded as insoluble at pH values between 6 and 8, and its solubility decreases with increasing molar mass. Detailed studies revealed that soluble cyanophycin fractions possess a higher lysine to arginine ratio relative to the insoluble form (Khlystov et al., 2017). The 3D structure of cyanophycin is not known, however, circular dichroism studies showed that under acidic conditions it exhibited a defined secondary structure containing β-sheets (Simon et al., 1980). Cyanophycin possesses negatively charged α-hydroxyl groups and positively charged arginine and lysine side chains, therefore, it is a zwitterion. Cyanophycin binds with other cyanophycin chains by charged interactions and hydrogen bonds. In addition, intra-chain interactions also occur.

Purified soluble cyanophycin is a white amorphous powder, while the purified insoluble fraction is a mixture of white and brownish solids. The reason for the occurrence of the brownish solids is unknown. Its mechanical properties are similar to other unplasticized polypeptides–it is rigid and brittle. According to previous studies (Khlystov et al., 2017) its Young’s modulus is 560 MPa, while the ultimate compressive strength is around 78 MPa and strain-at-break is 17 ± 4%. Cyanophycin is thermally stable up to 200°C, being pyrolyzed at > 700 C (Khlystov et al., 2017).

ε-Poly-l-lysine and γ-poly-glutamic acid are other examples of non-ribosomally synthesized polymers consisting of amino acids. They are examples of well-studied polymers, showing more than 3,000 hits for “poly-lysine” and more than 4,000 hits for “poly-glutamic acid” in a Web of Science search, compared to only 320 hits for “cyanophycin”. All have great potential for use in many diverse areas–environmental pollution treatments, food preservation and multiple medical applications (Chen et al., 2021; Nair et al., 2021).

Climate change is a fact (Heesterman, 2020). The European Commission prepared a strategy to slow down the destruction of the Earth (European Comission, 2020), stressing the adoption of a “less waste, more value” policy. Replacing polymers derived from fossil fuels with biodegradable biobased polymers is a crucial part of this strategy. Cyanophycin is a biobased polymer with a great potential for applications in many areas (Frommeyer et al., 2016), including in medicine (Uddin et al., 2020) and food technology (Sallam and Steinbüchel, 2010). Despite the fact that cyanophycin’s features do not allow this polymer to be used as a sole material, as for example polyhydroxyalkanoate (PHA), its biodegradability and environmentally friendly production encourages studies on widening its applicability. The availability of novel analogues of cyanophycin enables the determination of their features which may lead to the fulfillment of an industrial niche or the replacement of an existing but unsatisfactory solution. For instance, the modification of cyanophycin side chains with an alternative amino acid may not only yield novel polymers but may also lead to the production of dipeptides which could find other applications than aspartate-arginine (Sallam and Steinbüchel, 2010).

### Cyanophycin Production in Various Microorganisms and Plants

Cyanophycin serves as a storage compound for nitrogen and carbon in cyanobacteria (Li et al., 2001; Du et al., 2019). The first literature data on cyanophycin was published in 1971, describing the isolation and characterization of cyanophycin from *Anabaena cylindrica*, a nitrogen-fixing cyanobacterium (Simon, 1971). This was followed by the discovery of cyanophycin in many other cyanobacteria, such as *Nostoc ellipsosporum*, *Scytonema*, *Synechococcus* and *Synechocystis* and also in other microorganisms, for example *Acinetobacteria* and *Bordetella* species (Frommeyer et al., 2016; Du et al., 2019). The occurrence of cyanophycin in many microorganisms is discussed in a recent detailed review (Du et al., 2019).

Cyanobacteria are not suitable for the large scale production of cyanophycin because the growth of photosynthetic bacteria is slow and only low cell densities are obtained (Du et al., 2019). In addition, the photoautotrophic growth of cyanobacteria is only possible in photobioreactors. Thus heterologous expression of cyanophycin synthetase genes (*cphA*) in heterotrophic microorganisms was widely studied. The main sources of the genes were: *Synechocystis sp.* PCC6803 (*cphA*_*6803*_) (Frey et al., 2002), *Synechocystis sp.* PCC6308 (*cphA*_*6308*_) (Aboulmagd et al., 2001), and *Anabaena sp.* PCC7120 (*cphA*_*7120*_) (Voss et al., 2004a). Cyanophycin was heterologously produced for the first time in *E. coli* using *cphA* from *Synechocystis sp.* PCC6803. The genes were amplified from genomic DNA using PCR techniques and cloned into the pBluescript II SK + plasmid and transformed to *E. coli* strain DH5A. The yield of insoluble cyanophycin was around 100 mg/L (Ziegler et al., 1998).

Cyanophycin production at the 4.5 L scale was done with codon-optimized *cphA* gene using *E. coli* BL21 (DE3), obtaining 970 ± 80 mg/L cyanophycin (Khlystov et al., 2017). Large scale production was performed in a 500 L stirred-tank bioreactor. For this purpose *cphA* from *Synechocystis* sp. strain PCC6803 was expressed in *E. coli* DH1 on the pMa vector. The yield of cyanophycin biosynthesis achieved was 1.5 g/L (Frey et al., 2002). Cyanophycin synthetase from *Synechocystis sp*. strain PCC6308 was expressed in *E. coli* yielding a maximum cyanophycin content of 26.6% (w/w) of cell dry mass (Aboulmagd et al., 2000). In further studies, an addiction system was used to secure plasmid stability and applied to a recombinant *E. coli* HMS174(DE3) strain. This paid off with a cyanophycin content of 42% (w/w) (Kroll et al., 2011).

Other microorganisms have also been used for production of cyanophycin, for example *Ralstonia eutropha* and *Pseudomonas putida*, which are well established for PHA production (Voss et al., 2004b; Raberg et al., 2018). *P. putida* GPp104, *P. putida* KT2440, *R. eutropha* H16, *R. eutropha* PHB^−^4 carrying the *Anabaena sp.* strain PCC7120 cyanophycin synthetase were tested and the highest cyanophycin yield was obtained with *P. putida* KT2440 (Voss et al., 2004b). Interestingly, *R. euthropha* H16 possess in its genome genes homologous to cyanophycin synthetase. However, no cyanophycin was detected when these genes from *R. euthropha* H16 were both overexpressed in its natural host or heterologously expressed in *E. coli* (Adames et al., 2013).

*Corynebacterium glutamicum* is a bacterium that is used for the biotechnological production of various amino acids (Wendisch et al., 2016; Xiao et al., 2020). Therefore, it was investigated for the production of cyanophycin, however, with little success (2–3.6% of dry mass) (Aboulmagd et al., 2001). In addition the downstream process to isolate the polymer was difficult due to the rigidity of the cell walls (Aboulmagd et al., 2001). Other strains of *C. gutamicum* as well as various expression vectors and cyanophycin synthetases were later evaluated for this purpose with more success, resulting in cellular cyanophycin contents of approximately 14% of the dry mass (Wiefel et al., 2019b).

Yeast expression systems were also applied for cyanophycin synthesis. A particularly interesting idea was to use *Saccharomyces cerevisiae* to produce ethanol and cyanophycin simultaneously (Mooibroek et al., 2007; Könst et al., 2010). However, the yield of cyanophycin in *S. cerevisiae* was low in comparison to the *E. coli* system, amounting to only about 7% of the cellular dry mass (Steinle et al., 2008). Cyanophycin contents of up to 10.4% (wt/wt) were obtained when CphA_6308_ was expressed in *Pichia pastoris*. Truncation at the carboxy terminus led to a 2.5-fold higher specific activity of cyanophycin synthetase and 14.3% cyanophycin at the maximum (Steinle et al., 2010). Glutamic acid at the C-terminus of CphA_6308_ forms the M domain of the enzyme and its truncation probably led to conformational changes of the region thus causing the increase in activity.

In addition to microorganisms, transgenic plants were also constructed for cyanophycin production using agriculture. Two cultivars of *Nicotiana tabacum* carrying the *Thermosynecchococcus elongatus* BP-1 cyanophycin synthetase were used for this purpose yielding up to 9.4% dry weight cyanophycin (Nausch et al., 2016). Potato (*Solanum tuberosum*) is an alternative plant cyanophycin producer, however, the production yields were lower than that for tobacco cultivars, only amounting up to 7.5% dry weight (Schmidt et al., 2017). Although the yields are lower than for bacterial systems, it is arguable that plants are more suitable for the large scale production of cyanophycin due to their easier scalability and lower cost of cultivation (Nausch et al., 2015, 2016).

## Cyanophycin Modifications

The availability of novel structures of cyanophycin enables the determination of their features, which may lead to the fulfillment of an industrial niche or the replacement of an existing but unsatisfactory material. There are two principal strategies to change the composition of cyanophycin and to obtain new variants of this polymer. One strategy relies on the biological system and on the cultivation conditions used to grow the cyanophycin producing organism. This *in vivo* approach yields cyanophycin with a different composition which is then accumulated in the producing cells. The second strategy begins from cyanophycin which was produced *in vivo*, but is then isolated from the cells and subjected to post-synthetic modification by enzymes and/or chemicals.

### Changing Cyanophycin Composition *in vivo*

Cyanophycin from cyanobacteria is composed of nearly equimolar compositions of aspartic acid and arginine. Aspartic acid residues form the backbone of the polymer, while arginine residues are attached by their amino groups to the carboxyl group of each aspartate (Frommeyer et al., 2016; Du et al., 2019) (Figure 1A). However, the side chain of cyanophycin can be varied depending on the strain used for production and the conditions of cultivation, and most importantly, utilizing the low substrate specificity of most cyanophycin synthetases. For example, the purified CphA_MA19_ was able to introduce canavanine instead of arginine in the side chain (Hai et al., 2002), while CphA_29413_ incorporates a wider range of amino acids–lysine, ornithine, or citrulline (Berg et al., 2000; Du et al., 2019) (Figures 1A–E).

Lysine is an amino acid abundant in microbial cells and is more closely positioned on the metabolic map to the central metabolic pathways than arginine. It is therefore frequently added to cyanophycin chains at low concentrations in many microorganisms capable of synthesizing cyanophycin (Khlystov et al., 2017) (Figure 1B). *C. glutamicum* incorporates lysine into insoluble cyanophycin at around 5 mol%, while lysine is abundant in soluble cyanophycin, amounting to almost 50 mol%. The lysine content in cyanophycin influences its solubility. If its content is less than 4 mol%, cyanophycin does not become soluble even at 90 C, while cyanophycin with lysine contents above 31 mol% is soluble at 30 C (Wiefel and Steinbüchel, 2014). Amine groups on the lysine side chains form less hydrogen bonds than guanidine groups on arginine. In addition, lysine has a lower molecular weight than arginine, hence cyanophycin with an enhanced lysine content is more soluble (Khlystov et al., 2017).

Arginine can be replaced by glutamic acid in the side chain (Figure 1G) (Merritt et al., 1994; Wördemann et al., 2021). When *Synechocystis* sp. PCC 6308 was cultivated under nitrogen limiting conditions, no arginine was detected in cyanophycin, and instead glutamic acid was identified. The glutamic acid content decreased with increasing nitrogen supply (Merritt et al., 1994).

*C. glutamicum* developed for the production of target amino acids is able to incorporate lysine, citrulline ornithine and cadaverine into the side chain of cyanophycin, in particular while expressing the mutated CphA_6308_. The highest ornithine content was almost 12 mol%; the highest citrulline content was about 3 mol% (Wördemann et al., 2021) (Figure 1A–E). Interestingly, *C. glutamicum* can also incorporate glutamic acid in the backbone of the polymer (Figure 1H) (Wiefel et al., 2019b).

A *Pseudomonas putida* strain is able to form citrulline from arginine (Kakimoto et al., 1971) and was used for production of cyanophycin with a modified content. The recombinant strain was able to produce cyanophycin with about 8% citrulline when the CphA_6308_ cyanophycin synthetase was expressed. (Wiefel et al., 2011) (Figure 1C). Engineered *S. cerevisiae* strains expressing cyanobacterial CphA were able to accumulate cyanophycins with up to 20 mol% of citrulline (strain with deleted argininosuccinate synthetase), 8 mol% of ornithine (Figure 1E) (strain with ornithine carbamoyl transferase deleted) and 16 mol% of Lys (with argininosuccinate lyase deleted) (Steinle et al., 2009).

### *In vitro* Modifications of Cyanophycin

Beside *in vivo* variations of cyanophycin, the composition can also be varied *in vitro*. The composition of cyanophycin can be changed after its biosynthesis and subsequent isolation from the producing cells. For these post-synthetic modifications both enzymatic and chemical methods can be applied. Both strategies yield novel cyanophycin variants and thereby extend the number of available forms of cyanophycin.

#### Enzymatic Modifications

Enzymatic modifications of cyanophycin is a viable strategy to obtain new cyanophycin variants. The first modifications that should be mentioned is the use of cyanophycinases. These enzymes degrade the polymer and yield dipeptides. Cyanophycine derived dipeptides–mainly aspartate-arginine and aspartate-lysine–are highly bioavailable forms of amino acids. They have potentials for medical, nutrition or cosmetics applications (Sallam and Steinbüchel, 2010). Cyanophycinases are intracellular (CphB) or extracellular (CphE) enzymes. A bioinformatic analysis of bacterial strains revealed enzymes responsible for cyanophycin metabolism (Füser and Steinbüchel, 2007). Intracellular cyanophycinases occur in any bacterium accumulating cyanophycin in order to make the storage compound available to the cells again; extracellular cyanophycinases have also been found in various species including *Clostridium*, *Nitrosomonas*, *Nostoc*, *Pseudomonas*, *Ralstonia*, *Synechococcus* and others. These bacteria are able to use cyanophycin produced by other organisms if it is released from the decaying biomass (e.g., after a cyanobacteria blooming). A biotechnological process for the production of dipeptides using CphE from *Pseudomonas alcaligenes* has been developed (Sallam et al., 2009).

Arginine side chains of cyanophycin were treated with arginine deiminase (EC 3.5.3.15) from the European rabbit *Oryctolagus cuniculus* (Wiefel and Steinbüchel, 2016). This enzyme catalyzed reactions in which the imine group of arginine is converted to a ketone group. In nature this reaction occurs as a post-translational modification of peptide-bound arginine to citrulline (Figure 1C). The study demonstrated the possibility to modify the polymer enzymatically after its synthesis and to convert its side chain into citrulline.

#### Chemical Modifications

As mentioned above, cyanophycin can be hydrolyzed enzymatically by cyanophycinase. Chemical hydrolysis of this polymer was also studied, however, it requires much harsher conditions than its enzymatic counterpart. Acid hydrolysis yielded l-aspartic acid and l-arginine, while base hydrolysis allowed the release of l-arginine while polyaspartic acid is retained (Joentgen et al., 1998; Könst, 2011).

Lysine-rich cyanophycin produced in recombinant *E. coli* was subjected to treatment with *o*-methylisourea which yielded the guanidination of lysine residues that were converted to homoarginine (Figure 1F). The resulting polymer showed a similar solubility profile as insoluble cyanophycin and was soluble only at low or high pH. Such polymer can be degraded by CphE, yielding new dipeptides like aspartate-homoarginine (Frommeyer et al., 2014). Furthermore, it was proposed to introduce sulfur into lysine or arginine side chains, as it opens a path for further polymer modifications (Wiefel et al., 2019a). The reactions were conducted with methyl isocyanate and resulted in 50% conversion of lysine and 3% of arginine (Figure 1I). However, the yield of the reaction was much higher with dipeptides as substrates (72 and 96% respectively). N-acetylarginine and N-acetyllysine residues were obtained on the side chain of cyanophycin when the polymer was treated with acetic anhydride (Wiefel et al., 2019a) (Figure 1J). Cyanophycin was treated with formaldehyde to obtain a methylated version of the polymer (yielding a conversion of 84% for lysine and 15% for arginine residues) (Figure 1K). These modifications are expected to find applications in drug discovery research.

### Cyanophycin as a Component of Hybrid Materials

Cyanophycin is too brittle to be used alone as an individual material, as is also the case for other biobased polymers such as PHA or poly (lactic acid) (Boey et al., 2021). However, it could be applied as a component of copolymers due to its zwitterionic features, or conjugated with other polymers (Khlystov et al., 2017).

Cyanophycin was also investigated for use in biomedical applications. Insoluble cyanophycin was dissolved with HCl and cross-linked with glutaraldehyde. In these experiments thin films of a nearly smooth surface were obtained. The thickness of the film was proportional to the amount of cyanophycin. Prior to the cross-linking the cyanophycin films were brittle and fragile, while after cross-linking the films exhibited a structure of stacking lamellae, and were transparent and yellowish (Tseng et al., 2016).

Soluble cyanophycin was tested for toxicity to Chinese Hamster ovary cells. Minimal immune response was caused, no toxicity was observed, and, moreover, the cyanophycin films supplied adequate conditions for cell growth. Thus cyanophycin has the potential to serve as biocompatible and biodegradable material for biomedicinal applications (Tseng et al., 2016). Following these findings, cyanophycin was conjugated with polyethylene glycol (PEG) (Tseng et al., 2018). Two levels of conjugation were obtained: one with a low and one with a high level of PEG. The materials were prepared by reacting the aldehyde group of the activated PEG and the amine group of lysine in cyanophycin in the presence of sodium cyanoborohydride. The authors were investigating the thermoresponse of cyanophycin and its conjugates, aiming at unravelling its potential in drug delivery. The conjugate with a high PEG level proved to be a promising material for the temperature-regulated release of drugs.

In further research, cyanophycin was combined with two different polyanionic biopolymers, hyaluronic acid (HA) and γ-polyglutamic acid (PG), yielding polyelectrolyte multilayers. These were prepared by sequential adsorption of polyelectrolytes following the dip coat approach (Uddin et al., 2020). Both cyanophycin-PG and cyanophycin-HA films enabled growth, viability and enhanced cellular mobility when murine fibroblast cells were cultivated on them. Such features may be useful for tissue engineering.

## Conclusions and Perspectives

**FIGURE 2:**
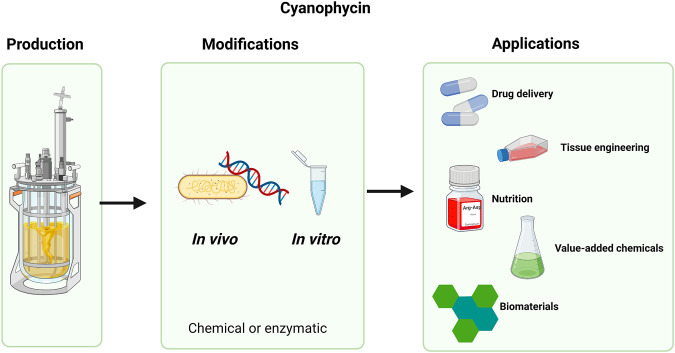
Flow chart presenting the cyanophycin life cycle. From microbial production, through chemical or enzymatic modifications to valuable applications.

To date, the major applications of cyanophycin have yet to be established. However, its modification widens its possible applications and opens new perspectives (Figure 2). Khlystov et al., 2017 thoroughly characterized material properties of cyanophycin and suggested its usefulness as part of copolymers–cyanophycin could contribute to the final material with its strength. For example, cyanophycin could be used to toughen resins. Moreover, Tseng et al., 2016 showed that cross-linking cyanophycin led to decrease of brittleness, hence, this technique may also widen the applicability of cyanophycin.

The selective hydrolysis of cyanophycin can result in its conversion to polyaspartic acid, which can be used for the production of detergents, dishwasher soaps, as well as in biomedical applications (Du et al., 2019; Adelnia et al., 2021). Enzymatic degradation of cyanophycin leads to the formation of various dipeptides, mainly Asp-Arg or Asp-Lys. It could in the future also yield other dipeptides if the corresponding polymers are available in sufficient amounts. Dipeptides are bioavailable sources of amino acids. l-aspartic acid, l-arginine and l-lysine are used in numerous specimens for the treatment or prevention of various conditions, such as endocrine, cardiovascular or genitourinary disorders. The treatment of patients with both arginine and aspartate were reported to help to lower cholesterol levels, enhance wound healing, decrease erectile disfunction, and other beneficial results. Arginine and lysine are important in human and animal nutrition as their presence is correlated to cardiovascular functioning and the release of growth hormone (Sallam and Steinbüchel, 2010).

Cyanophycin can be included in the design of drugs and tissue engineering, as was recently demonstrated (Tseng et al., 2018; Uddin et al., 2020). Chemical and enzymatic modifications of cyanophycin allow the introduction of new functional groups into the polymer, thus expanding the area of possible applications (Wiefel et al., 2019a).

Another interesting proposed application focused on the concept of a cyanophycin biorefinery. Engineered *S. cerevisiae* would produce ethanol and cyanophycin simultaneously, subsequently the cyanophycin would be enzymatically or chemically hydrolyzed and converted to value-added chemicals. Cyanophycin hydrolysis yields arginine and aspartic acid. Using l-arginase (EC 3.5.3.1) the arginine of cyanophycin could first be converted to l-ornithine, and then l-ornithine could be a substrate for the production of other chemicals, for example nylon. This activity of l-arginase was shown only with free arginine (Könst et al., 2010). Similarly, aspartic acid could be converted to industrially important chemicals. For example, l-Aspartate α-decarboxylase (EC 4.1.1.11) catalyzes the reaction of aspartic acid with β-alanine, which could be further converted to acrylamide or acetonitrile (Könst, 2011).

The material properties of modified cyanophycin are not known. Most of the aforementioned modified polymers were not obtained in quantities large enough to perform full characterization. With enhanced yields of the modification reactions, sufficient amounts of products can be synthesized and tested. Consequently, new attractive applications may be found.
